# Workplace violence and influencing factors among paramedic pre hospital paramedic personnel (city and road) in Iran: a quality content analysis

**DOI:** 10.1186/s12873-021-00520-5

**Published:** 2021-10-29

**Authors:** Marziye Hadian, Alireza Jabbari, Hojjat Sheikhbardsiri

**Affiliations:** 1 Health Services Management, Health Management and Economics Research Center, Isfahan University of Medical Sciences, Isfahan, Iran; 2 Health in Disasters and Emergencies Research Center, Institute for Futures Studies in Health, Kerman University of Medical Sciences, Kerman, Iran

**Keywords:** Violent behavior, Pre-hospital emergency, Paramedic, Health system, Iran

## Abstract

**Background:**

The goal of every emergency department is to provide the highest quality services in the shortest time using limited resources. However, occupational violence is so prevalent among pre-hospital paramedic personnel that some experts claim that it is impossible to find pre-hospital personnel without an experience of violence in the workplace. Therefore, it seems necessary to investigate the causes of violence among this population group and find ways to control it.

**Aim:**

The present study aimed to investigate the Violence and influencing factors among paramedic pre-hospital personnel.

**Method:**

This qualitative study was conducted to explore the views of a group of pre-hospital paramedic personnel (*n* = 45) selected through purposive sampling. The data was collected through in-depth and semi-structured interviews and analyzed using Graneheim and Lundman’s conventional content analysis methods. The trial version of MAXQDA 16 software was used to manage the coding process.

**Results:**

Based on the results of the analysis of data collected from prehospital paramedic personnel, three main categories including: human factors, organizational factors, and environmental factors and 20 subcategories were detected.

**Conclusion:**

If authorities neglect violence in the workplace and do not take serious actions to prevent it, violence and, more importantly, “hostility” will gradually prevail in the workplace. It also increases the stress and anxiety of staff and consequently severely deteriorates their job performance. Hence, authorities are strongly recommended not to ignore this issue and, instead, take measures, for instance hold workshops, to train personnel about the techniques of anger and violence control.

## Introduction

The goal of every emergency department is to provide the highest quality services in the shortest time using limited resources [1]. Nearly four decades have passed since introducing emergency medicine as one of the medical specialties, and today it is presented as one of the leading and well known fields in medical assistant programs [2]. Pre hospital system is a sensitive and important part of medicine and various types of traffic accidents, occupational accidents, poisonings, injuries during quarrels or fights, as well as other dangerous situations caused by some diseases are considered as important causes of emergencies in patients [3].

Job is a part of everyone’s life and employees often experience different emotions in their workplace. For example, the occurrence of conflict or violence in the workplace (such as verbal conflict with patients or their relatives in the emergency room) often causes negative emotions in staff [4–6]. In general, “violence” is a behavior that a person commits against another one, leading to physical, sexual, psychological, cultural, and economic harm [5, 7].

In the emergency department and at the time of medical emergencies, there are two types of violence or aggression: the first type is the aggression of injured people or their relatives against emergency personnel and the second type is violence and aggression of emergency personnel against injured people or others [8]. In the first type, the main source of violence is patients, victims, or their relatives. These people have no personal relationship with emergency personnel (i.e. they do not already know them).

As one of the main causes, the violence perpetrated by victims or their relatives is attributed to the intense stress or anxiety they are experiencing at that moment [6]. According to the US Department of Labor, in three-quarters of cases of workplace violence, the abuser has no personal connection with staff [9]. However, in the second type of violence, the source of violence is the people working in medical departments or emergency wards who show violence against victims or their relatives [4, 10]. It is worth noting that when violence is committed by employees, no disciplinary action is usually taken, because such a violence is attributed to staffs’ anger or troubles caused by issues in their workplace [4, 11]. Nonetheless, being upset and angry cannot justify committing violence and aggression in the workplace, because it can have detrimental consequences for the organization [12]. Violent encounters generate negative energy in the workplace and prevent people from proper job performance [11, 13].

### Literature review

Maguire et al., studied identify the risks and factors associated with work-related physical violence against Emergency Medicine Services (EMS) personnel internationally and showed that Overall, men experienced more assaults than women, and younger workers experienced more assaults than older workers [5]. Sahebi et al. studied prevalence of workplace violence types against personnel of prehospital in Iran through a systematic review study and reported the prevalence of physical, verbal, and cultural workplace violence among EMS personnel was 36.39% [14]. Benjamin et al., in India studied workplace violence among prehospital care providers and showed that the overall prevalence of any workplace violence was 67.9%. The prevalence of physical violence and verbal violence was 58 and 59.8%, respectively [15].

### Aim of study

Occupational violence is so prevalent among prehospital paramedic personnel that some experts claim that it is impossible to find an prehospital personnel without an experience of violence in the workplace [9]. Therefore, it seems necessary to investigate the causes of this violence and find ways to control it. Hence, this study was aimed at investigating the causes of violence among prehospital paramedic (city or road) personnel. The results of this study can help community health authorities and managers of the health services organization reduce violence against prehospital paramedical personnel thorough promoting good relationship between prehospital paramedical personnel and people of the community including patients and their families.

## Methods

This qualitative study was conducted from January 2019 to November 2020 and used content analysis methods. Content analysis can be used with qualitative or quantitative data, induction or deduction. This method is useful when the existing theory or research literature on a certain phenomenon is limited [16].

### Setting, participants and data collection

This qualitative study was conducted in Iran and the study population was pre hospital paramedic personnel in Tehran, Isfahan, Shiraz and Mashhad. The number of participants in this study included 45 nurses and technicians. A total of 27 males and 18 females were interviewed, ranging from 25 to 44 years of age and approximately 10 people from each province were selected for interview. Among the 45 participants in the study, 21 nurses and 24 technicians. Participants were chosen using a purposeful sampling method. Inclusion criteria consisted of participants with a bachelor’s degree in nursing or an associate degree or bachelor’s degree in prehospital emergency nursing, at least 1 year of work experience in a prehospital emergency center, and their willingness to participate in the study; exclusion criteria included lack of consent for participating in the study. Sampling was carried out until data saturation occurred, i.e. when the researcher concluded that further interview would fail to provide new information.

This qualitative study was done using in-depth and semi structured interviews beginning with open questions, gradually continuing more detailed. Interviews began with broad questions such as “Please describe your experiences with violence against patients?” “What were the causes of this violence most of the time?” “What was the most common cause of such behavior on your part?” “Would you explain more?” Probing was done according to the reflections of each participants about experiences of violence against pre-hospital emergency personnel. The interviews were taped and lasted from 25 to 74 min. Time and place of interviews was set by agreement between the researcher and older adults. Field notes were written during interviews to describe and interpret the responses correctly. The interviews were conducted by H.S.H. face-to-face and individually. The process of data collection was under the supervision of D.A.J.

### Data analysis

The data gathered in this stage were analyzed by the content analysis approach using Graneheim and Lundman method [17]. In this inductive process, the researcher reads the transcribed texts several times in order to fully understand them. Next, the units of meaning (words, sentences or paragraphs) that answered questions about workplace violence and influencing factors were selected, condensed and labeled with a code. Similar codes which represented similar concepts were classified into subcategories and then made into a category (manifest level). Each category emerged from a group of content that shared a commonality, so the categories were internally homogeneous and externally heterogeneous. The relationship between the underlying meanings in categories, which is an expression of the latent meaning, emerged as the main theme. The trial version of the MAX QDA 16 Software was used to manage the coding process.

### Reliability and validity

This study employed strategies recommended by Lincoln and Guba for reliability and validity tests [18]. According to this recommendation, four criteria of creditability, dependency, conformability, and transferability are required to ensure reliability. To increase data creditability, the study researchers engaged with data and the environment for 9 months while constantly making observations and compiling field notes. Dependency of data was assessed by peer check strategies. Peer check was performed on a monthly basis so to ensure that the research team had a thorough discussion about emerged data. Background information and personal interest of researchers on the corresponding topics and document maintenance were used for assessing conformability of data. The context of the interviews, codes, and the extracted categories were reviewed by the research team and other professional colleagues in the field of qualitative research. Using sampling with maximum variation, the researchers were able to collect quite a mixed variety of different comments, observations, and interpretations.

## Results

### Demographic of participants

The participants included 45 nurses and paramedic technicians with the mean age of 34.5 ± 4.8 years, ranging from 25 to 44 years. The mean duration of work experience was 13.25 ± 3.4 years and all the participants had more than 2 years of experience in EMS.

## Main results

Based on the results of data analysis, a total of 20 subcategories were obtained. After several rounds of reviewing and summarizing the data and taking into account similarities and differences, three main categories were obtained through the content analysis method, including 1) Human Factors, 2) Organizational factors, and 3) Environmental factors; the results are also presented in Table 1. The three major categories were in turn classified into several sub-categories, extracted via analyzing hand-written notes and interviews. The categories and corresponding sub-categories are described in the following sections.

**Table 1:** Themes and sub-themes of factors related to violent behavior in Prehospital paramedic personnel (city or road)

Factors related to employee violence	
Theme	Subtheme
Human factors	1. Fatigue 2. Stress 3. Job dissatisfaction 4. Anxiety Disorders 5. Family issues 6. Loss of confidence 7. Fear 8. Conflict with negative thoughts
Organizational factors	1. Dissatisfaction with the work environment 2. large number of shifts 3. Lack of equipment 4. Lack of levelization 5. Unclear job description 6. Lack of constructive communication with colleagues 7. Ambiguity in tasks or overlap between job tasks
Environmental factors	1. Occurrence of work accidents 2. People’s ignorance 3. Traffic problems 4. Unreasonable expectations of people

### Human factors involved in the incidence of violence in employees

According to the findings, most of the challenges were among the interviewees in this area. Based on most of the interviewees, excessive fatigue, stress, job dissatisfaction, anxiety disorders, family problems, loss of self-confidence, fear, and conflict with negative thoughts are among the human challenges involved in the incidence of violence among pre-hospital emergency staff.

*“Someone who wants to work in Emergency 115 must be very strong. Because they are confronted with scenes that, for example, they do not know the person in front of them committed suicide, hit by a car, and .... Too often, many employees experience depression and fear in their lives because of this, and these characteristics lead to inappropriate behavior.” (Interviewee No. 1).*

*“Sometimes personal issues and problems put so much pressure on us, so that our behavior becomes really involuntary. The fact that people do not understand exacerbates this problem. Many times, these problems are caused due to our job, but there is no solution.” (Interviewee No. 12).*

*“Now it is not right to keep talking about violence. Many times, patients’ companions are really violent against us. Their behavior makes us to feel stressed and anxious and we are forced to show a series of behaviors.” (Interviewee No. 9).*

“*Anyone who wants to work in Emergency 115 must have a strong spirit. Having negative thoughts and lack self-confidence for work make the person to suffer from stress and aggression.” (Interviewee No. 21).*

*“Sometimes our situation is difficult, the facilities are few, the street conditions are awful; then the patient’s surroundings cause fear and stress in us; with their behavior, which causes unpleasant behaviors.” (Interviewee No. 40).*

### Organizational factors involved in the incidence of violence in employees

According to most of the interviewees, dissatisfaction with the work environment, large number of shift, lack of equipment, lack of levelization, unclear job description, lack of constructive communication with colleagues, ambiguity in tasks or overlap between job tasks are among the organizational challenges involved in the incidence of violence among pre-hospital emergency staff.

**“***We work so hard in terms of work shifts; our situation is really complicated. They reduce payments for overtime. His mind is obsessed, then he tells himself that he is not receiving the items he deserves. They do not give him the benefits they should, as compared with someone who works in a hospital. That is why they are subconsciously discouraged. They get bored and may argue with the patient***.”** *(Interviewee No. 31).*

*“Sometimes there are so many things to do, and I get really confused about doing them. I feel very tired because of this confusion that occurs elsewhere.” (Interviewee No. 33).*

*“Another issue is the financial discrimination between us and the hospital staff. We do not receive the benefits they receive in the hospital. Our work shifts are more than their work shifts. We work longer hours, but we get paid less.” (Interviewee No. 6).*

*“Our problem is the lack of an independent organization. It would be great if we had an independent organization. Because here, in addition to the pressure and stress of work, we must also have the stress and worry that at any moment we may be told to leave here.” (Interviewee No. 42).*

*“ Many situation are in the form of canopies. Metal sheds are cold in winter and warm in summer. We do not have gas. We are very annoyed. If we want to get out, we have to turn on the heater, which may burn everything. Inside ambulances, there is little equipment for critical situations. For example, in the face of Corona virus epidemic, we could hardly provide services.” (Interviewee No. 33).*

*“ There is no organizational line for us and we will leave the system after a few years. This thought is constantly with us and affects our behavior.” (Interviewee No. 35).*

### Environmental factors involved in the incidence of violence in employees

According to most of the interviewees, occurrence of work accidents, people’s ignorance, traffic problems, unreasonable expectations of people, and unclear job description are among the environmental challenges involved in the incidence of violence among pre-hospital emergency staff.

*“ One of the things that makes our colleagues angry, and may be very obvious, is street traffic that makes us late in most cases. This delay causes the anger of those around the patient. Because we ourselves are in a bad situation, we can no longer tolerate their bad behaviors and react. “(Interviewee No. 29).*

*“One thing I have to point out is people’s misplaced expectations. People contact us for every details. Now, talking about coronavirus, we are sometimes contacted for a small cough. Sometimes they are annoying us on the phone. For example, we had a case where a call was made and we went to the place and saw that it was called because of a very simple weakness.” (Interviewee No. 17).*

*“When something happens in a place, everyone becomes an expert. They make nonsense comments. They interfere in our work. work environments are crowded and these behaviors really affect us.” (Interviewee No. 45).*

## Discussion

In general, “violence” is a behavior that a person commits against another one, causing physical, sexual, psychological, cultural, and economic harm [12]. Severe physical injuries usually have visible physical symptoms, such as bruising, fractures, tears, bleeding, burns, etc. However, behaviors such as shouting, humiliating, breaking things, or throwing objects as a sign of anger and psychological trauma in general do not have vivid physical symptoms, while they strongly affect mental health [19]. In this study, the causes of violence among pre-hospital emergency staff were investigated. In this study, the causes of violence were classified into three categories: human, organizational, and environmental factors.

### Human factors

According to the findings of the study, human factors include extreme fatigue, stress, job dissatisfaction, anxiety disorders, family problems, loss of confidence, fear, and conflict with negative thoughts. Prehospital paramedic personnel, due to the special condition of the patient, fear of incapacity in saving a patient’s life, time constraints, etc.

In Bayrami et al.’s study that was conducted on “Mashhad pre-hospital emergency challenges”, the findings showed that stress and loss of self-confidence were the main causes of violence [20]; it is consistent with the results of the present study.

The findings of another study by Dadashzade showed that job dissatisfaction was one of the factors affecting the behavior of employees in emergency centers.

In addition, according to the results of the mentioned study, patients or their companions violence against personnel and lack of a clear legal and protection system for dealing with violence against personnel are some of the challenges faced by personnel; it is in line with the results of the present study [11]. In another study by Moradi et al., the findings showed that excessive workload, job insecurity, and job depression led to burnout and inappropriate behaviors [8]. The results of Moradi’s study confirm the results of the present study.

### Organizational factors

According to the findings of this study, structural factors including dissatisfaction with work environment, large number of shifts, lack of equipment, lack of levelization, unclear job descriptions, lack of constructive communication with colleagues, ambiguity in tasks or overlap between job tasks are important causes of dissatisfaction and violence in EMS personnel. The finding of a study by Bahrami et al. showed that 85.6% of emergency stations complained about large number of shifts. A study in West Azerbaijan by Vali et al. also showed that 66% of emergency personnel were dissatisfied with the lack of job descriptions and 45.63% of employees were willing to leave their jobs due to lack of levelization [21].

Considering the structural challenges of pre-hospital emergencies, a number of challenges are related to equipment such as lack of medical supplies, worn-out ambulances, and improper arrangement of facilities that hinder the provision of quality services. In Vatankhah et al.’s study conducted on pre-hospital emergency challenges, the findings show that one of the most important challenges faced by pre-hospital emergency staff is the lack of resources and depreciation. The results of Vatankhah et al.’s study is consistent with the results of the present study [22]. In a study by Erie et al., independence of Emergency and multiplicity of commands were among the challenges affecting the delivery of EMS care services [23]. The results of the present study are in line with the findings of the mentioned study.

### Environmental factors

According to the findings of this study, environmental factors including accidents, people’s ignorance, traffic problems, and unreasonable expectations of people are important factors involved in the incidence of dissatisfaction and stress and violent behaviors among pre-hospital emergency staff. The findings of a study by Torabi et al., showed that unpredictable conditions at the scene, late arrival at the scene, and the resulting stress lead to stress in pre-hospital emergency personnel [2]. The findings of another study by Eri et al., showed that route conditions and traffic problems were important factors affecting Emergency staff [23]. Furthermore, the findings of Jamshidi et al.’s study, showed that the very low participation of organizations such as traffic police led to consequently and, to severe distrust, which is consistent with the results of the present study [24]. Moreover, in Hosseinikia et al.’s study, EMS staffs’ dissatisfaction with improper expectations of people and their ignorance were the causes of dissatisfaction [10]; it is consistent with the results of the present study.

In general, cognitive processes related to occupational violence can be explained within six stages. Step 1: The staff encounters an event or events, such as a conflict with one of the relatives of the injured persons, making the staffs anxious and putting a lot of stress on them.

Step 2: The employee thinks the problem is unsolvable. Step 3: While expressing regret over the situation, the employee places all the responsibilities on others and does not blame himself at all.

Step 4: The employee confronts and rejects every process that indicates he or she is also involved in creating current problems. At this stage, the staff convinces himself that the situation is not his fault and at the same time, he does not think of a peaceful solution to get out of that situation. Step 5: The only solution that comes to mind is violence. The staff thinks that he or she can use violence to bring things to an end. Step 6: The employee commits a violent behavior or at least tries to show it. However, if staff learn anger and aggression control skills, they can easily prevent this recurring cycle [3, 6, 25].

### Limitations

Due to the fact that this study was conducted in only four provinces, to reach more accurate conclusions, it is better to conduct the study as a surveillance at the national level.

## Conclusion

Violence has many negative consequences. In most cases, these negative consequences or effects are not limited to the workplace, and may extend to the community level. If authorities remain passive against the violence in the workplace and do not take serious actions to prevent it, violence and, more importantly, “hostility” will gradually prevail in workplace. It also increases the stress and anxiety of the staff and consequently severely deteriorates their job performance. Hence, authorities are strongly recommended not to ignore this issue easily and, instead, take measures, for instance hold workshops, to train personnel about the techniques of anger and violence control. The results of this study can help community health authorities and managers of the health services organization reduce violence against prehospital paramedical personnel thorough promoting good relationship between prehospital paramedical personnel and people of the community including patients and their families. Also results of this study also suggest that in prehospital system with create organizational rows, regulation of laws and administrative regulations reform in relation to the range of services offered by emergency personnel, attention to the facilities and cooperation and the participation of other organizations, such as media and traffic to enhance pre-hospital emergency services at the community level can be effective in reducing the challenges in hospital emergency system.

## Data Availability

The data sets generated during the current study are available from the corresponding author.
